# Life-Course Transitions to Precarious Housing in Older Age

**DOI:** 10.1093/geroni/igaa057.2005

**Published:** 2020-12-16

**Authors:** Helen Barrie, Debbie Faulkner, Laurence Lester

**Affiliations:** 1 University of South Australia, Adelaide, South Australia, Australia; 2 The Australian Alliance for Social Enterprise, University of South Australia, Adelaide, South Australia, Australia

## Abstract

Home is central to health and wellbeing; yet the changing nature of work, household dynamics and especially housing markets, with scant policy attention and action around this, means low-middle income households are struggling in many countries. In Australia, while older people are considered to be at less risk because of higher levels of home ownership, there is a growing body of evidence about the living situations of older people who have not attained or retained home ownership over the life course and have limited wealth and savings moving into later life. This paper presents the findings of multivariate regression modelling using HILDA, a national longitudinal panel survey, to identify the profile(s) of older people at risk of homelessness in Australia. The data makes it clear a range of structural and individual factors across the life course are increasingly impacting on the ability to live a good life in older age.

